# An Investigation of Precise Orbit and Clock Products for BDS-3 from Different Analysis Centers

**DOI:** 10.3390/s21051596

**Published:** 2021-02-25

**Authors:** Pengli Shen, Fang Cheng, Xiaochun Lu, Xia Xiao, Yulong Ge

**Affiliations:** 1 National Time Service Center, Chinese Academy of Sciences, Xi’an 710600, China; chengfang@ntsc.ac.cn (F.C.); luxc@ntsc.ac.cn (X.L.); xiaoxia@ntsc.ac.cn (X.X.); geyulong15@mails.ucas.ac.cn (Y.G.); 2 University of Chinese Academy of Sciences, Beijing 100049, China; 3 Key Laboratory of Precise Positioning and Timing Technology, Chinese Academy of Sciences, Xi’an 710600, China

**Keywords:** BDS-3, tropospheric delay, positioning performance, precise point positioning (PPP), orbit comparison, clock differences, analysis centers

## Abstract

A quality evaluation of precise products for BDS-3 constellations is presented for the first time in this contribution. Then, the tropospheric delay retrieval and positioning performance of BDS-3 precise point positioning (PPP) solutions using the precise products (gbm, wum, iac, sha, cnt) with observations from 24 stations from DOY 280 to 317 in 2020 was comprehensively investigated. The orbit comparisons present consistencies of 0.09–0.22 m for the C19–C37 satellites and of 0.5–1.2 m for the C38–C46 satellites among the final products. The standard deviation (STD) values of the clock differences of iac showed the best agreement with those of gbm, followed by wum, sha. The clock differences performance of cnt was the worst. For BDS-3 PPP solutions with five Analysis centers (ACs) products, the median convergence times of static PPP mode incorporating the gbm, wum, iac, sha, and cnt products were 31.0, 33.5, 34.5, 37.8, and 72.0 min, respectively; the median convergence times of kinematic PPP model incorporating the same products were 40.5, 41.0, 50.5, 55.0, and 94.0 min, respectively. The positioning accuracies in the static and kinematic modes were approximately 1–4 cm, 2–6 cm in the horizontal and vertical components, respectively. With the final products in kinematic mode, the performance of PPP with real-time products (cnt) is poorer than all PPP with final products. The median of ZTD accuracies of the five products gbm, wum, iac, sha, and cnt were 7.84, 7.58, 7.04, 7.19, and 10.1 mm, respectively, and the accuracy differences were very small among five AC products (gbm, wum, iac, sha).

## 1. Introduction

The global BeiDou navigation system (BDS-3) is the third generation navigation satellite system of China. It is a global positioning, navigation, and timing (PNT) service system following the international standard and providing compatibility and interoperability with other global navigation satellite systems (GNSSs). BDS-3 has been completely implemented since 31 July 2020. The BDS-3 constellations consist of three geostationary Earth orbit (GEO), three inclined geosynchronous orbit (IGSO) and 24 medium Earth orbit (MEO) satellites [1]. For global positioning users, B1I, B3I, B1C, B2a [2], and B2b [3] signals will be transmitted. For regional users, in the Asia-Pacific region, B2a is applied for satellite-based augmentation system (SBAS), and B2b is utilized for precise point positioning (PPP).

Thanks to the precise GNSS clock and orbit products released by the International GNSS Service (IGS) [4] and the Multi-GNSS Experiment (MGEX) [5], PPP has already been widely applied in various services and applications, such as positioning [6], navigation, timing [7,8], geodesy, atmosphere monitoring [9,10], and low Earth orbit (LEO) satellite orbit determination [11]. However, the accuracy and reliability of PPP performance is mainly based on the quality of the clock and orbit products employed by the users. Hence, it is particularly important that efforts should be made to improve satellite clock and orbit products [12].

The commonly used precise products are provided by the MGEX and IGS Analysis centers (ACs) [5,13]. In recent studies, the PNT performances with use different precise products were compared and investigated by some researchers [12,14,15,16,17]. Zhou et al. [12] and Guo [18] mainly focused on multi-GNSS PPP performance with different products and evaluated precise products released by the MGEX and IGS ACs. PPP time and frequency transfer were studied by Zhang and Tu [16] based on different satellites and precise products, and the results indicated that the PPP time transfer with the precise products from Center for Orbit Determination in Europe (CODE) and GeoForschungsZentrum (GFZ) exhibited better performance than those using other products. In another previous investigation, the precise products from European Space Agency’s Space Operations Centre (ESA/ESOC) were adopted by some researchers for GPS + GLONASS PPP processing [19]. The precise products from GFZ or WUM were preferred by some other researchers for multi-GNSS PPP processing [20,21,22]. However, previous studies have mostly focused on PPP performance using the precise products of GPS, GLONASS, Galileo, BDS-2, and a comprehensive investigation on the performance of BDS-3 PPP in tropospheric delay retrieval and positioning using different precise products remains unclear.

Hence, this study mainly focuses on evaluating the quality of different products, the tropospheric delay retrieval in 24-h observation sessions and positioning performance analysis in 6-h observation sessions, as calculated from BDS-3 PPP using different precise products (gbm, wum, iac, sha, cnt). The positioning performance was evaluated in kinematic and static PPP modes, while the tropospheric delays were studied using PPP with fixed coordinates. The tropospheric delay retrieval and positioning performances were carried out using observations from 24 stations from Day of year (DOY) 280 to 317 in 2020.

This paper is organized as follows: an overview of the MGEX precise products are introduced in Section 2, and the modeling of the coordinate parameters and the GNSS IF PPP observation model are described in Section 3. Afterwards, the dataset and processing strategies are described in Section 4. In Section 5, the performances of orbit/clock comparisons, PPP solutions, and ZTD estimation with various precise products are performed and compared. The mainly findings are concluded in Section 6.

## 2. Introduction of MGEX Precise Products

Currently, multi-GNSS products for MGEX are provided by eight ACs, as follows:

(1) Center for Orbit Determination in Europe (CODE), Switzerland.
(2) Centre National d’Etudes Spatiales and Collecte Localisation Satellites (CNES/CLS), France.
(3) Wuhan University (WHU), China.
(4) Deutsches GeoForschungs Zentrum (GFZ), Germany.
(5) Japan Aerospace Exploration Agency (JAXA), Japan.
(6) Technische Universität München (TUM), Germany.
(7) Shanghai Astronomical Observatory (SHAO), Chinese Academy of Sciences, China.
(8) Information and Analysis Center (IAC), Russia.

The precise orbit and clock products provided by various ACs are listed in Table 1. In the interest of brevity, ‘‘wum’’, ‘‘gbm’’, ‘‘iac’’, ‘‘sha’’, “com’’, ‘‘jax’’ and ‘‘tum’’ denote precise products from WHU, GFZ, IAC, SHAO, CODE, JAXA and TUM, respectively. In addition, the real-time multi-GNSS products (cnt) are provided by CNES/CLS, and can be downloaded from http://www.ppp-wizard.net/products/REAL_TIME/ (accessed on 1 December 2020).

Note that CODE, TUM and JAXA are excluded in this work due to lack of BDS-3 products. Hence, GFZ, WHU, IAC, SHAO, and CNES/CLS, are chosen in our work.

## 3. Methods

The methods applied to assess the precision and accuracy of the orbit and clock products are introduced in this section, and the IF PPP model together with two coordinate parameters modeling are then described. The positioning performances were evaluated by using static and kinematic modes, while coordinate-fixed mode will be utilized for ZTD estimation.

### 3.1. Comparisons among Precise Products

Consistency among different precise products is usually applied for evaluations of precise products quality [23]. In this study, comparison of orbit products were presented in the Radial-, along-, cross-track (RAC) directions at 15-min intervals. Then, the three-dimensional (3D) orbit consistency was calculated. The clocks were aligned to a reference satellite by differencing to remove systematic biases and then compared at 5-min intervals.

Due to the different stagey of quality control among various precise products, outliers with extremely large differences are inevitable. We regarded outliers as a phenomenon in which the clock time series or orbit differences were three times larger than the median value; these differences were excluded. The root-mean-square (RMS) of orbit differences and the standard deviation (STD) of the clock differences were utilized to assess the quality of the precise products.

### 3.2. IF PPP Model

The IF pseudorange and carrier phase observation equations can be written as follows [24]:

(1) $$P_{IF}^{s}=\rho_{}^{s}+e_{r}^{s}\cdot \delta X_{r}+c\delta t_{r,IF}-c\delta t^{s}+B_{r,IF}-B_{IF}^{s}+M_{w}T_{w}$$

(2) $$\Phi_{IF}^{s}=\rho_{}^{s}+e_{r}^{s}\cdot \delta X_{r}+c\delta t_{r,IF}-c\delta t^{s}+\lambda_{IF}N_{IF}+b_{r,IF}-b_{IF}^{s}+M_{w}T_{w}^{}$$

where *r* and *s* indicate the receiver and satellite, respectively; Φ is the carrier phase observations; *λ_IF_* indicates the wavelength of the carrier phase; $e_{r}^{s}$ is the unit vector from the receiver to the satellite; *δ***X***_r_* is the vector of the coordinate increments; *B_r,IF_* and $B_{IF}^{s}$ represent receiver and satellite uncalibrated code delays (UCDs); *c* is the speed of light; *δ**t**_r,IF_* is the receiver clock offset; *δt^s^* denotes the satellite clock offset; *b_r,IF_* and $b_{IF}^{s}$ indicate the receiver and satellite uncalibrated phase delays (UPDs); *N_IF_* denotes integer ambiguity; *M_w_* indicates the wet mapping function; *T_w_* refers to the tropospheric zenith wet delay (ZWD). Note that the B1I/B3I IF combination was employed to analyse PPP performance.

In our work, PPP with static and kinematic modes were applied. In the static PPP mode, the coordinate parameters are regarded as a constant, as follows:

(3) $$X(h)=\hat{X}(h-1)$$

where $\hat{X}(h-1)$ is the estimated position; *h* indicates the epoch number.

In the kinematic model, the position parameters are usually estimated as white noise model, as follows:

(4) $$X(h)\sim N(0,\sigma^{2})$$

## 4. Experimental Setup

First the experimental setup, including observation data from MGEX stations, is described in this section. Then, the distribution of selected stations and the data processing strategies are described.

### 4.1. Dataset

**Figure 1:**
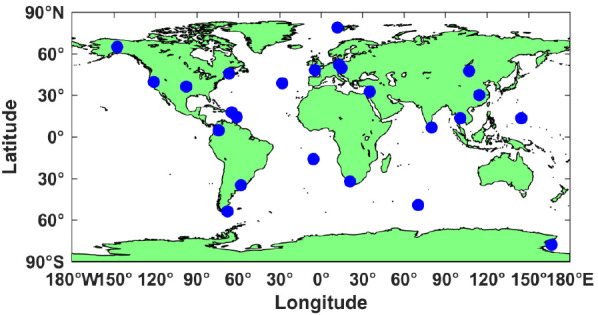
Geographical distribution of the 24 selected MGEX stations.

Observations from 24 MGEX stations were selected with a sample interval of 30 s from DOY 280–317 in 2020. All observed stations can track all BDS-3 satellites. The distribution of all the selected stations is presented in Figure 1.

### 4.2. Processing Strategy

In this study, data processing was based on an ionospheric-free PPP solution, and all PPP tests were investigated using the developed GAMP [25]. The eliminated errors and models applied in this contribution were listed in Table 2.

The orbit/clock error are corrected by the precise products. The first-order ionospheric delay was removed and mitigated by the ionosphere-free (IF) mode. Note that the phase center offsets (PCOs), phase center variations (PCVs), Sagnac effect, relativistic effects, tidal loadings, and phase windup were mitigated according to the corresponding models [29] for clarity. The Zenith hydrostatic delays (ZHD) are corrected by the Saastamoinen model [27]. The ambiguities were estimated as constant. The IGS weekly solutions in Solution Independent Exchange (SINEX) format were set as the true coordinates. In addition, the final ZTD daily solutions from CODE were applied as the true values.

## 5. Result and Analysis

The results of the orbit/clock comparisons are first introduced in this section. Then, the positioning performances of static and kinematic PPP modes and ZTD estimation were compared and assessed with the different precise products. Boxplot representing the 25th to 75th percentiles (boxes) with the median (50th percentile) and whiskers representing the 5th and 95th percentiles were employed by Scanlon et al. [30] to evaluate the PPP convergence performance and ZTD accuracy.

### 5.1. Orbit Comparisons

**Figure 2:**
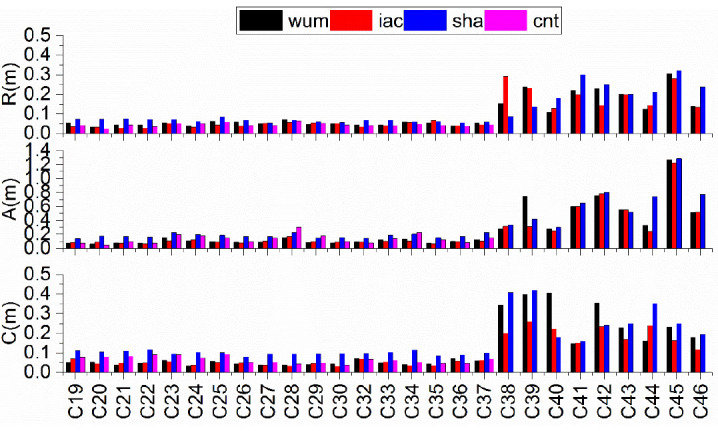
Orbit comparisons of precise BDS-3 products of MGEX ACs with respect to gbm.

Considering the characteristics of BDS-3 constellation, the orbit accuracy was assessed using individual satellites. The differences in the RAC components with respect to gbm are illustrated in Figure 2, and Table 3 gives the constellation-averaged 3D RMS of the orbit comparisons. As shown in Figure 2 and Table 3, for the C19–37 satellites, the orbit differences show the best consistency, while the C38–46 satellites show poor performance. The reason for this fact is that few MGEX stations can track the C38–46 satellites.

For a given satellite orbit, the differences in the R-track component is smaller than those in the A-track and C-track components. The 3D RMS values of orbit differences of the wum, iac, sha, and cnt products were 0.1213, 0.1175, 0.2158, and 0.1599 m, respectively for the C19–37 satellites and 0.7143, 1.2780, 0.7912, and -m, respectively for the C38–47 satellites. The RMS values of the differences with respect to the gbm, wum, and iac products are basically equivalent, while sha shows the worst RMS among the final products. Interestingly, cnt is slightly better than sha, which can be explained by the fact that the precise orbit products released by sha show the worst agreement with that of gbm. Overall, for the C19–37 satellites, iac and wum exhibited the best consistency at the 10-cm level, and the orbit differences showed an agreement of 0.09–0.22 m among the ACs, while for the C38–46 satellites, the quality of orbit differences was worse than that of the C19–37 satellites, and the orbital 3D RMS values are mostly within 0.5 and 1.2 m.

### 5.2. Clock Comparisons

**Figure 3:**
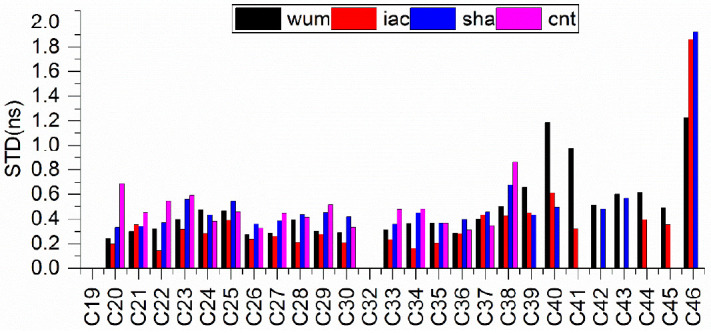
Clock comparisons of the precise BDS-3 products of MGEX ACs with respect to gbm.

Figure 3 presents the STD of the individual satellite clock differences with respect to gbm, and the STD of different types are given in Table 4. Combining Figure 3 and Table 4, three findings can be obtained. First, for the C19–37 satellites, the STD of clock differences showed better agreement respect to gbm, the STD of the clock differences report a consistency of 0.1–0.7 ns, whereas the STD of the clock differences of the C38–46 satellites is approximately 0.3–2.0 ns. This phenomenon is comparable with that of the orbit performances. As we mentioned previously, the reason is that there are few MGEX stations that support C38–46 satellites. Second, it was found that the STD of the clock differences of iac showed the best agreement with that of gbm, followed by wum and sha, the STD of the clock differences performance of cnt was worst. The reason for this result is that the real-time multi-GNSS products (cnt) are estimated using Kalman filter. Third, more interestingly, for the C19–37 satellites, we find that iac and wum show the best consistency of 0.15 ns, and they report a consistency of the STD of the BDS-3 clock differences among five ACs at the 0.15–0.5 ns level. We see that the STD of the clock differences of wum, iac, sha and cnt with respect to gbm are 0.3361, 0.2627, 0.3814 and 0.4258 ns, respectively, for the C19–37 satellites and 0.6518, 0.4911, 0.5676, and 0.4140 ns, respectively, for the C38–47 satellites.

### 5.3. Static PPP Solutions

**Figure 4:**
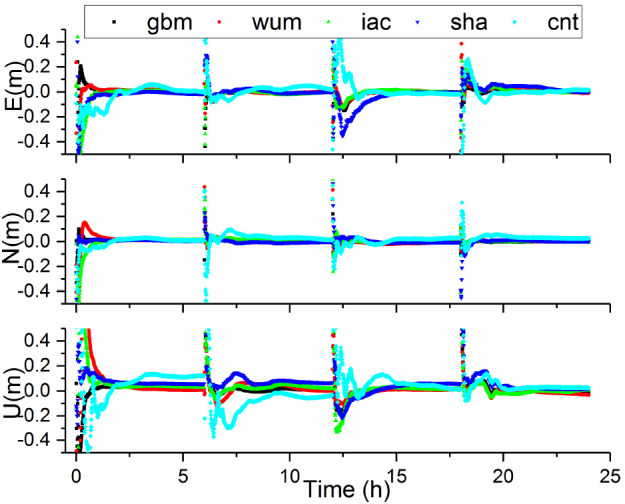
Positioning errors obtained from BDS-3 PPP in static mode with different products on DOY 288 at CUSV.

Note that “convergence” was defined as a situation meeting the conditions under which 3D positioning errors at the current epoch and at the following epochs were within 0.1 m. In order to obtain more experimental results, we refer to the method in the paper [31] and divide the 24-h observation sessions into four every 6-h observation sessions for calculation. The positioning errors of BDS-3 PPP [32,33,34] for station CUSV, with five AC products on DOY 288, are displayed Figure 4. An in-depth investigation into the presented numerical values raises a number of interesting findings. First, we can see that the positioning accuracies in static PPP solutions are basically equivalent. Furthermore, the convergence time of PPP with cnt products is the longest. One possible explanation for this result may be that the accuracy of real-time products is relatively poor and the real-time products can only support the C19–C37 satellites. Additionally, the convergence time of the sha products was poor among the final products. This is reasonable since the accuracies of the orbit and clock differences of the sha products were relatively worse than those of other products.

**Figure 5:**
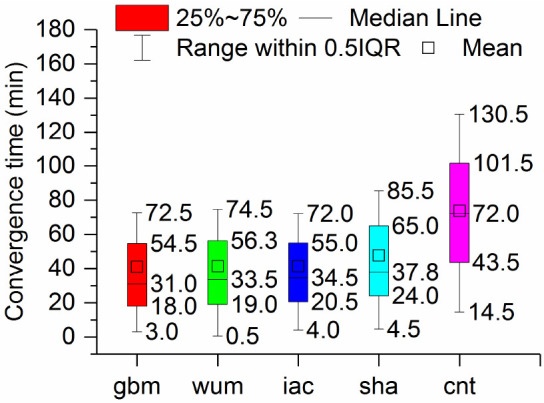
Boxplot of the convergence time calculated from BDS-3 static PPP with five precise products on DOY 288.

To further quantify our conclusions, Figure 5 indicates the boxplot of the convergence time calculated from BDS-3 static PPP with five precise products (gbm, wum, iac, sha, cnt). The median, 25th percentile and 75th percentile of the convergence time of the five products of the five products, gbm, wum, iac, sha, and cnt, are 31.0, 33.5, 34.5, 37.8, and 72.0 min, 18.0, 19.0, 20.5, 24.0, and 43.5 min, and 54.5, 56.3, 55.0, 65.0, and 101.5 min, respectively. Overall, the static PPP performance with the gbm provided by GFZ ranked the first in convergence time. In addition, the PPP performance with the wum products was slightly better than that of the iac products. PPP with the sha products performed worst in the final products. Moreover, the convergence time of PPP with cnt was poorer than those of PPP solutions with final products.

**Figure 6:**
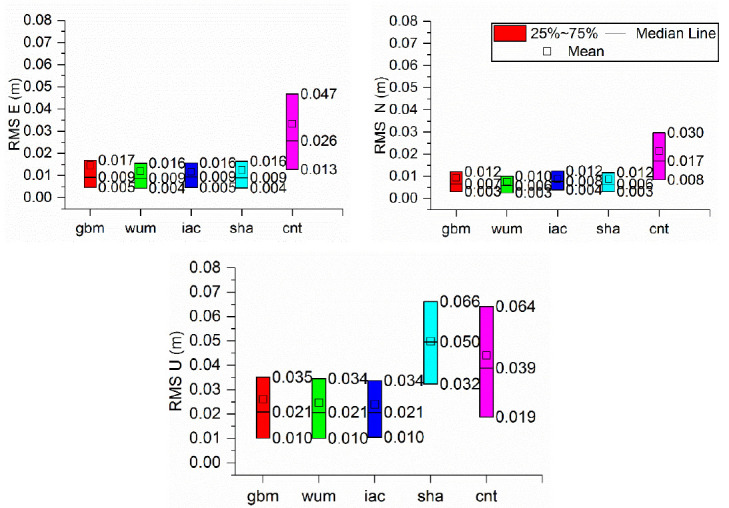
Boxplot of the positioning accuracy in East, north, and up (ENU) directions derived from BDS-3 PPP in static mode with different precise products.

In addition to the convergence time, the positioning accuracy was also evaluated in the static model. Figure 6 indicates the boxplot of the positioning accuracy in East, north, and up direction derived from BDS-3 PPP in static mode with different precise products. The monthly median, 25th percentile and 75th percentile positioning accuracies calculated from BDS-3 PPP solutions with different precise products are summarized in Table 5, Table 6 and Table 7, respectively. Similar to the results obtained for the convergence time, the positioning accuracies with sha and cnt are lower than those with the gbm, wum and iac products. The monthly median positioning accuracies with gbm, wum and iac were 0.009, 0.007, and 0.021 m in ENU directions, respectively, while those using the sha and cnt products were 0.010, 0.008, and 0.040 m and 0.026, 0.017, and 0.050 m, respectively. The monthly 25th percentile of the positioning accuracies with gbm, wum and iac were 0.005, 0.003, and 0.010 m in the ENU directions, respectively, and the monthly 75th percentile of the positioning accuracies with gbm, wum and iac were 0.016, 0.012, and 0.034 m in the ENU directions, respectively. The 25th percentile positioning accuracies obtained using sha were 0.006, 0.004, and 0.019 m in the ENU directions, respectively, and those in the 75th percentile for the same products were 0.013, 0.008, and 0.032 m in the ENU directions, respectively. The 25th percentile of the positioning accuracies of the cnt products were 0.028, 0.012, and 0.064 m in the ENU directions, respectively, and those in the 75th percentile were 0.047, 0.030, and 0.066 m in the ENU directions, respectively.. From the median positioning accuracy, the differences among the five precise products were less than 1 mm for the gbm, wum and iac products in static PPP solutions. Overall, the positioning accuracies are approximately 1 and 4 cm in the horizontal and vertical components, respectively, for the final products in static mode. In addition, the PPP performance with real-time products had much room for growth.

### 5.4. Kinematic PPP Solutions

**Figure 7:**
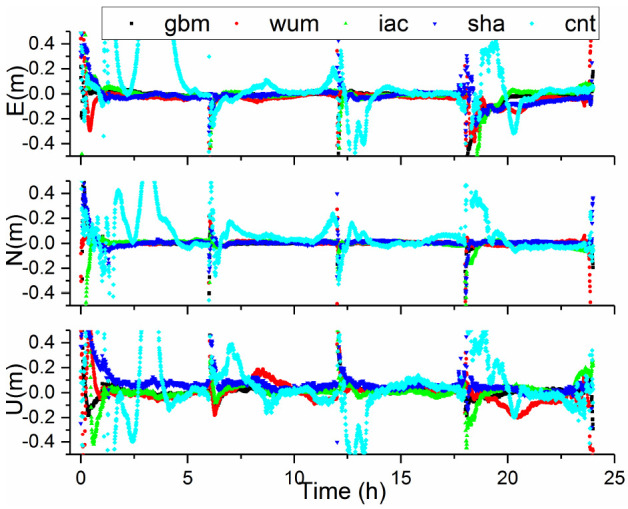
Positioning errors obtained from BDS-3 PPP in kinematic mode with different products on DOY 288 at CUSV.

In this subsection, “convergence” is defined as a situation meeting the condition that the 3D positioning errors at the current epoch and at the following epochs were within 0.2 m. In order to obtain more experimental results, we apply the same method in the static mode PPP solutions to divide the 24-h observation sessions into four every 6-h observation sessions for calculation. The positioning errors of BDS-3 PPP in kinematic model for the CUSV station, with five AC products on DOY 288, are displayed in Figure 7. Three interesting conclusions can be found. First, we can see that the positioning accuracies in kinematic PPP solutions are basically equivalent to the final products after convergence. Second, the kinematic PPP mode with cnt products had the worst performance among five products. This fact can be explained by two reasons. One is that the accuracy of the product is relatively poor. Another reason is that only the C19–C37 satellites are incorporated in the cnt products. Finally, similar to the performance of the static mode, the convergence time of PPP with cnt products was the longest, the reason for this result is same as that mentioned above for the static mode.

**Figure 8:**
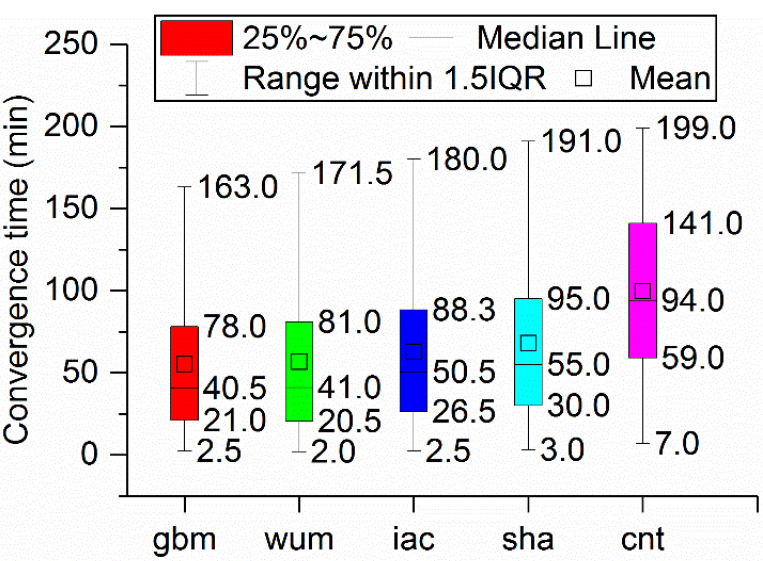
Boxplot of the convergence time of BDS-3 kinematic PPP with precise products from five ACs.

Figure 8 gives a boxplot of the convergence time obtained from BDS-3 PPP in kinematic mode with five precise products (gbm, wum, iac, sha, and cnt). It is apparent that the median the convergence time of the five products, gbm, wum, iac, sha, and cnt, were 40.5, 41.0, 50.5, 55.0, and 94.0) min, respectively; the 25th percentile values were 21.0, 20.5, 26.5, 30.0, and 59.0 min, respectively; and the 75th percentile values were 78.0, 81.0, 88.3, 95.0, and 141.0 min, respectively. Obviously, the convergence performance of the kinematic PPP solution with the gbm products provided by GFZ demonstrated the best performance, with the wum products following. Moreover, the PPP performance with the wum products was slightly better than that of the iac products. PPP with sha products performed worst in the final products.

**Figure 9:**
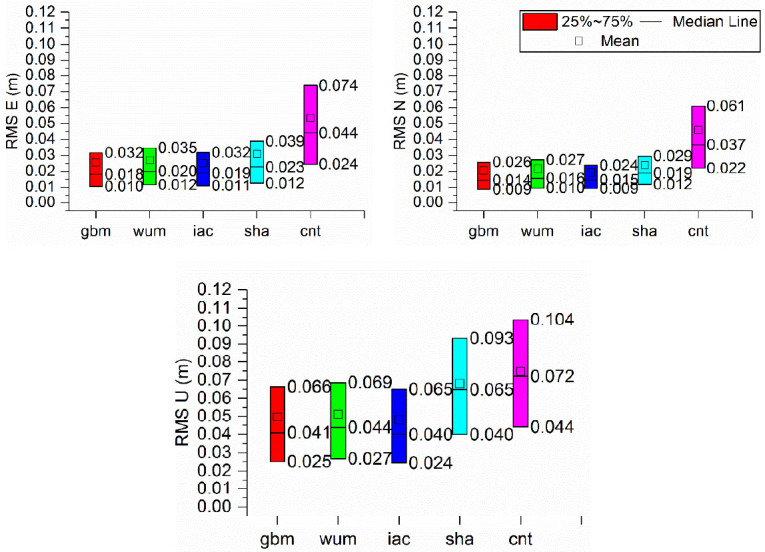
Boxplot of the positioning accuracy in ENU directions derived from BDS-3 PPP in Kinematic mode with different precise products.

In addition, the positioning accuracy derived from kinematic model was evaluated. Figure 9 indicates the boxplot of the positioning accuracy in ENU directions derived from BDS-3 PPP in kinematic mode with different precise products. Table 8, Table 9 and Table 10 give monthly median, 25th percentile and 75th percentile values of the positioning accuracies of BDS-3 PPP solutions with five precise products, respectively. As we can see from the tables, the PPP accuracies with the sha and cnt products were generally lower than those with the gbm, wum and iac. The monthly median positioning accuracies obtained with the gbm, wum and iac products were (0.018, 0.014, 0.041), (0.020,0.016,0.044), (0.019, 0.015, 0.040) m at ENU directions, respectively, while those obtained with sha and cnt products were (0.023, 0.019, 0.065) m and (0.044, 0.037, 0.072) m. Note that the monthly 25th values of the positioning accuracies with gbm, wum, iac and sha show comparable performances of 0.011, 0.012, and 0.025 m in the ENU directions, respectively, and the monthly 75th percentile values were 0.032, 0.026, and 0.065 m in the ENU directions, respectively. For the median values of the positioning accuracies, the differences were within 4 mm for the gbm, wum and iac products in kinematic PPPs. Additionally, the positioning accuracies were approximately 2 and 6 cm in the horizontal and vertical components, respectively, with final products in kinematic mode.

### 5.5. ZTD Estimation

**Figure 10:**
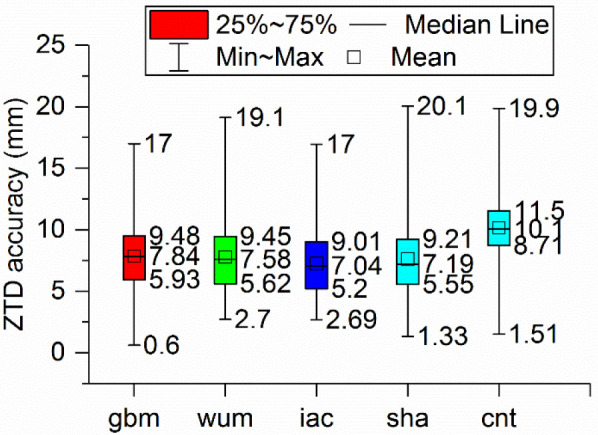
Boxplot of the ZTD accuracy derived from BDS-3 PPP in coordinate-fixed mode with five precise products.

The fixed BDS-3 PPP solutions with the precise products were applied to derive ZTD, and comparisons of the derived ZTD respect to CODE was performed. Figure 10 illustrates the distribution of the accuracies of the ZTD obtained through BDS-3 PPP solutions with five AC products. Notably, the median ZTD accuracies of the five products, gbm, wum, iac, sha, and cnt, were 7.84, 7.58, 7.04, 7.19, and 10.1 mm, respectively; the 25th percentile values of the same products were 5.93, 5.62, 5.2, 5.55, and 8.71 mm, respectively; and the 75th percentile values were 9.48, 9.45, 9.01, 9.21, and 11.5 mm, respectively.. It is apparent that differences in median, 25th percentile and 75th percentile of ZTD accuracy among the products (gbm, wum, iac, sha) were less than 1 mm. Moreover, the ZTD accuracies derived from coordinate-fixed BDS-3 PPP mode with cnt products demonstrated the worst quality. Similar to the static PPP, the ZTD accuracies were not strongly correlated with performance of the satellite clock and orbit products. Overall, the ZTD accuracies obtained through BDS-3 PPP solutions with the different final products showed the similar performance.

## 6. Conclusions

An assessment of the quality of BDS3 precise clock and orbit products was first made through comparisons among the MGEX ACs (gbm, wum, iac, sha, and cnt). Then, the contributions of the five MGEX (gbm, wum, iac, sha, and cnt) precise products on the BDS-3 positioning performance and the ZTD derived from BDS-3 PPP solutions were comprehensively evaluated based on observations from DOY 280–317 in 2020 of 24 MGEX stations that can track BDS-3 signals. It is very significant that during PPP processing with the five AC products, the processing settings and software were the same; hence, the only variable was the precise BDS-3 clock and orbit products. Four main findings can be concluded:

First, the 3D RMS values of orbit differences for the wum, iac, sha, and cnt products were 0.1213, 0.1175, 0.2158, and 0.1599 m, respectively, for the C19–37 satellites and 0.7143, 1.2780, 0.7912, and -m, respectively, for the C38–47 satellites with respect to gbm. the performance of the BDS-3 orbit comparisons indicated a consistency of 0.09–0.22 m for the C19–C37 satellites, and a consistency of 0.5–1.2 for the C38–C46 satellites among the final products.

Second, for the C19–37 satellites, the STD of the clock differences showed the better agreement with respect to gbm, and the STD of the clock differences reported a consistency of 0.1–0.7 ns, whereas the STD of clock differences of the C38–46 satellites were approximately 0.3–2.0 ns. The STD of the clock differences for the wum, iac, sha, cnt products were 0.3361, 0.2627, 0.3814, and 0.4258 ns, respectively, for the C19–37 satellites and 0.6518, 0.4911, 0.5676, and 0.4140 ns, respectively, for the C38–47 satellites with respect to gbm. The STD of the clock differences of iac products showed the best agreement with those of gbm products, followed by wum, sha. The performance of cnt products was the worst.

Third, in static mode, the median of the convergence times for the implementation of BDS-3 PPP with the gbm, wum, iac, sha, and cnt products were 31.0, 33.5, 34.5, 37.8, and 72.0 min, respectively. The monthly median positioning accuracies obtained with the gbm, wum and iac products were 0.009, 0.007, and 0.021m in ENU directions, respectively, while those obtained using sha products were 0.010, 0.008, and 0.040 m in ENU directions, respectively, and those obtained using cnt products were 0.026, 0.017, and 0.050 m, respectively. In the kinematic mode, the median of the convergence time of the gbm, wum, iac, sha, and cnt were 40.5, 41.0, 50.5, 55.0, and 94.0 min, respectively. The monthly median positioning accuracies obtained with gbm, wum and iac were 0.018, 0.014, and 0.041 m in ENU directions, respectively; and those obtained with iac were 0.019, 0.015, and 0.040 m in the same directions, respectively. The monthly median positioning accuracies obtained using sha were 0.023, 0.019, and 0.065 m in the ENU directions, respectively, and those obtained using cnt were 0.044, 0.037, and 0.072 m in the same directions, respectively.

Finally, for the ZTD estimation, the median of ZTD accuracy of the gbm, wum, iac, sha, cnt products were 7.84, 7.58, 7.04, 7.19, and 10.1 mm, respectively. The differences of ZTD accuracy were very small among the MGEX AC products (gbm, wum, iac, sha), while the accuracy obtained with cnt products showed a much worse performance.

## Figures and Tables

**Table 1:** Information of precise products and MGEX ACs.

Institution	Prefix	System	Orbit/Clock
GFZ	gbm	GRCEJ	5 min/30 s
WHU	wum	GRCEJ	15 min/30 s
IAC	iac	GRCEJ	5 min/30 s
SHAO	sha	GRCE	5 min/5 min
CNES/CLS	cnt	GRCE	5 min/5 s
CODE	com	GRCEJ	5 min/30 s
JAXA	jax	GRJ	5 min/30 s
TUM	tum	CEJ	5 min/5 min

**Table 2:** Models used in data preprocessing.

Items	Processing Strategies
Estimator	Kalman filter
Observations	IF observables
Signals	B1I + B3I
Sampling interval	30 s
Cutoff angle	10°
Phase wind-up	Corrected [26]
Tropospheric delay	ZWD: estimated, GMF mapping function [27] ZHD: corrected
Relativistic effect	Corrected
Tidal displacement	IERS Conventions 2010 [28]
Sagnac effect	Corrected [28]
PCO and PCV	Corrected by “atx” file
Station coordinates	Estimated as constant
Receiver clock offset	Estimated as white noise
Phase ambiguities	Estimated as constant

**Table 3:** 3D RMS values from comparisons of precise BDS-3 orbit products among MGEX ACs (units: m).

	wum		iac		sha		cnt		gbm	
	C19–37	C38–46	C19–37	C38–46	C19–37	C38–46	C19–37	C38–46	C19–37	C38–46
**wum**	-	-	0.0909	0.4838	0.2034	0.5337	0.1382	-	0.1213	0.7143
**iac**			-	-	0.1953	0.5177	0.1346	-	0.1175	1.2780
**sha**					-	-	0.2027	-	0.2158	0.7912
**cnt**							-	-	0.1599	-
**gbm**									-	-

**Table 4:** STD of the BDS-3 precise clock differences among five products (units: ns).

	wum		iac		sha		cnt		gbm	
	C19–37	C38–46	C19–37	C38–46	C19–37	C38–46	C19–37	C38–46	C19–37	C38–46
**wum**	-	-	0.1448	0.5145	0.2344	0.4952	0.2979	0.3304	0.3361	0.6518
**iac**			-	-	0.2590	0.4698	0.3044	0.2844	0.2627	0.4911
**sha**					-	-	0.3343	0.3333	0.3814	0.5676
**cnt**							-	-	0.4258	0.4140
**gbm**									-	-

**Table 5:** The median of positioning accuracy in ENU directions derived from BDS-3 PPP in static mode with different precise products (unit: m).

	Static PPP		
	E	N	U
gbm	0.009	0.007	0.021
wum	0.009	0.006	0.021
iac	0.009	0.008	0.021
sha	0.010	0.008	0.040
cnt	0.026	0.017	0.050

**Table 6:** The 25th percentile of positioning accuracy (unit: m) in ENU directions derived from BDS-3 PPP in static mode with five precise products.

	Static PPP		
	E	N	U
gbm	0.005	0.003	0.010
wum	0.004	0.004	0.010
iac	0.005	0.003	0.010
sha	0.006	0.004	0.019
cnt	0.013	0.008	0.032

**Table 7:** The 75th percentile of positioning accuracy (unit: m) in ENU directions derived from BDS-3 PPP in static mode with different precise products.

	Static PPP		
	E	N	U
gbm	0.017	0.012	0.035
wum	0.016	0.010	0.034
iac	0.016	0.012	0.034
sha	0.028	00012	0.064
cnt	0.047	0.030	0.066

**Table 8:** The median of the positioning accuracy (unit: m) in the ENU directions calculated from BDS-3 Kinematic PPP with different precise products.

	Kinematic PPP		
	E	N	U
gbm	0.018	0.014	0.041
wum	0.020	0.016	0.044
iac	0.019	0.015	0.040
sha	0.023	0.019	0.065
cnt	0.044	0.037	0.072

**Table 9:** The 25th percentile of the positioning accuracy (unit: m) in the ENU directions derived from BDS-3 kinematic PPP with five precise products.

	Kinematic PPP		
	E	N	U
gbm	0.010	0.009	0.025
wum	0.012	0.016	0.027
iac	0.011	0.009	0.024
sha	0.012	0.012	0.040
cnt	0.024	0.022	0.044

**Table 10:** The 75th percentile of the positioning accuracy (unit: m) in the ENU directions derived from BDS-3 Kinematic PPP solutions with five precise products.

	Kinematic PPP		
	E	N	U
gbm	0.032	0.026	0.066
wum	0.035	0.027	0.069
iac	0.032	0.024	0.065
sha	0.039	0.029	0.065
cnt	0.074	0.061	0.072

## Data Availability

All data can be available from Multi-GNSS Experiment (MGEX).
